# Prevalence of metabolically unhealthy obesity, overweight, and normal weight and the associated risk factors in a southern coastal region, Iran (the PERSIAN cohort study): a cross-sectional study

**DOI:** 10.1186/s12889-021-12107-7

**Published:** 2021-11-05

**Authors:** Ghazal Zoghi, Roghayeh Shahbazi, Masoumeh Mahmoodi, Azim Nejatizadeh, Masoumeh Kheirandish

**Affiliations:** 1 Cardiovascular Research Center, Hormozgan University of Medical Sciences, Bandar Abbas, Iran; 2 Cellular and Molecular Medicine Department, Faculty of Medicine, University of Ottawa, Ottawa, Canada; 3 Endocrinology and Metabolism Research Center, Hormozgan University of Medical Sciences, Bandar Abbas, Iran; 4 Molecular Medicine Research Center, Hormozgan Health Institute, Hormozgan University of Medical Sciences, Bandar Abbas, Iran

**Keywords:** Metabolic health, Obesity, Prevalence, Prospective Epidemiological Research Studies in IrAN (PERSIAN)

## Abstract

**Background:**

Obesity is a substantial risk factor for cardiovascular and metabolic diseases. Epidemiologic studies have shown that some obese and overweight individuals are metabolically healthy. We aimed to determine the prevalence of metabolically unhealthy obesity (MUO), metabolically unhealthy overweight (MUOW), and metabolically unhealthy normal weight (MUNW) in a southern coastal area of Iran, Bandare-Kong Non-Communicable Diseases (BKNCD) Cohort Study.

**Methods:**

This population-based study included the participants of BKNCD, as part of the Prospective Epidemiological Research Studies in IrAN (PERSIAN). Metabolic health was defined as not fulfilling the metabolic syndrome (MetS) criteria.

**Results:**

Among the 3917 participants in this study with the mean age of 48.29 ± 9.39 years, including 1691 (43.2%) males, the age- and sex-standardized prevalence of MUO, MUOW, and MUNW was 13.9, 16.8, and 6.4%, respectively. Binary logistic regression analysis revealed that the adjusted odds of all metabolically unhealthy states were higher in older age groups, except for MUO whose adjusted odds were lower in the 65–70 age group compared to the 55–64 age group. Illiteracy was significantly correlated with MUOW (adjusted OR: 1.43, 95% CI 1.09–1.87, *P* = 0.010); however, it was not associated with MUO or MUNW. Higher body mass index (BMI) was significantly correlated with MUNW but it was not associated with MUOW or MUO. Higher waist circumference (WC) was also significantly associated with all metabolically unhealthy states.

**Conclusion:**

The age- and sex-standardized prevalence of MUO, MUOW, and MUNW was 13.9, 16.8, and 6.4%, respectively in the current study. Advanced age and higher WC were significantly correlated with all metabolically unhealthy states, while illiteracy and higher BMI were only associated with MUOW and MUNW, respectively. Metabolic health rather than weight loss should be the focus and objective of public prevention programs.

## Introduction

Although obesity is a major concern worldwide and epidemiological evidence suggests that obesity is an independent risk factor for many diseases including type 2 diabetes mellitus, cardiovascular diseases, nonalcoholic fatty liver disease, sleep apnea, hypertension, stroke, and several types of cancer [1–3], some individuals are metabolically healthy despite being obese. This is called metabolically healthy obesity (MHO), which is defined by the absence of metabolic abnormalities, such as dyslipidemia, hyperglycemia, and hypertension in individuals with body mass index (BMI) within the obesity range [4–6]. It has been reported that mild weight gain does not necessarily cause metabolic dysfunction when individuals are otherwise metabolically healthy. Similarly, weight loss may not necessarily decrease cardiometabolic risk in individuals with MHO and may even result in a contradictory response (e.g. a decline in insulin sensitivity instead of the expected increase) [7, 8]. Surprisingly, adipose tissue may protect against the adverse effects of metabolic syndrome [9].

The prevalence of MHO varies widely (10–40% of all the obese); nevertheless, age, ethnicity, and geography do not account for such variety. Most definitions of MHO are based on the absence of metabolic syndrome (MetS) or some of its individual components [10]. Lack of a standard definition for metabolic health can be responsible for this disparity. Yet, in a collaborative analysis of 10 large cohort studies across Europe, MHO prevalence was reported 7–28% in women and 2–19% in men, despite using a harmonized MHO definition; obesity without any components of MetS and no previous cardiovascular disease diagnosis [11].

There is another concept referred to as metabolically unhealthy obesity (MUO); in spite of similar levels of total excess body fat compared to those with MHO, the consequences of obesity have already developed in individuals with MUO [12–14]. Conversely, a cluster of metabolic abnormalities exists in many normal-weight individuals. These are referred to as metabolically unhealthy normal weight (MUNW), whose cardiovascular events and all-cause mortality appear to be comparable to those with MUO [2, 15, 16].

Metabolic health seems to be more important than normal weight in preventing future cardiovascular events, with the metabolically healthy obese having similar outcomes compared to metabolically healthy normal weight individuals. Besides, most of the studies on these concepts lack two categories of individuals, namely metabolically healthy overweight (MHOW) and metabolically unhealthy overweight (MUOW). Therefore, in the current study we aimed to investigate the prevalence of MUNW, MUOW, and MUO and the associated risk factors in the PERSIAN Bandare-Kong Cohort Study.

## Methods

### Participants and study design

In this cross-sectional study, we evaluated participants of the PERSIAN Bandare-Kong Cohort Study, as part of the Prospective Epidemiological Research Studies in IrAN (PERSIAN) which has been previously described in detail [17]. The cohort study includes 4063 individuals aged 35–70 years, recruited between November 17, 2016 and November 22, 2018 from Hormozgan province, southern Iran. After exclusion of incomplete records and pregnant women, the baseline data of 3917 individuals were included in the final analysis of the current study.

### Data collection

Face-to-face interviews were done by trained interviewers in order to collect sociodemographic data, including age, education, marital status, occupation, place of residence, and cigarette smoking. The cumulative calorie content of the daily ingested foods was also recorded as “daily calorie intake” using the food frequency questionnaire (FFQ) validated for the Iranian population [17]. For estimation of daily calorie intake, the portions of ingested foods were converted into gram weight multiplied by the energy/calorie content of each type of food per gram weight. Metabolic equivalent of tasks (METs) was used to report weekly energy expenditure.

A standard digital scale with measurement accuracy of 0.5 kg was used to weigh the subjects while they wore minimum clothing and no shoes. Height was measured with normally set shoulders while subjects were standing shoeless. Waist circumference (WC) was measured at the end of several normal breaths, at a level parallel to the floor, the midpoint between the top of the iliac crest and the inferior margin of the last palpable rib in the midaxillary line. WC was measured twice for each participant and the average was recorded. The largest circumference of the buttocks, at a parallel level to the floor, was measured as hip circumference (HC). All measurements were done with the same stretch-resistant tape to the nearest 0.5 cm. Subjects were standing upright during the measurements, with arms relaxed at the side, feet evenly spread apart, and body weight evenly distributed. Waist-to-hip ratio (WHR) was calculated as WC divided by HC to the nearest 0.01. BMI was calculated as weight in kilograms divided by the square of the person’s height in meters to the nearest 0.01. According to the WHO guidelines, BMI < 25 kg/m^2^ was regarded as normal, 25 ≤ BMI < 30 as overweight, and BMI ≥30 as obese [18].

A standard mercury sphygmomanometer with an appropriate for arm circumference was used to measure blood pressure (BP) after 5 min of rest, in the seated position, with feet on the floor, and arm at heart level. The average of two measurements made at least 5 min apart was recorded.

Following overnight fasting, 5-ml venous blood samples were collected and fasting plasma glucose (FPG) was measured using the glucose oxidase method. Also, triglyceride (TG) and high-density lipoprotein (HDL) were measured for each participant using the enzymatic method. The details of sample collection and storage, as well as measurements have previously been published [17].

The International Physical Activity Questionnaire was used for the assessment of physical activity [19]. The weekly average of 24-h physical activities including leisure time activities, work, and exercise was categorized into 3 groups, namely low physical activity 24–36.5 METs, moderate (36.6–44.9 METs), and high (≥45 METs) [20]. The wealth index, an estimation of the socioeconomic status, has previously been explained in detail. Based on this score, participants were graded and then classified into tertiles, including poor, average, and rich [21]. WHR was classified into low, moderate, and high according to the World Health Organization (WHO) separate cut-offs for men and women [22].

MHO was defined as BMI ≥30 kg/m^2^ and not having MetS according to the criteria proposed by Meigs et al. [23] and Aguilar-Salinas et al. [24]. The National Cholesterol Education Program Adult Treatment Panel III (NCEP-ATP III) criteria for MetS [25], along with cut-offs for WC replaced by ≥95 cm in both men and women, was used as the Iranian-specific criteria [26, 27]. MHOW was defined as 25 ≤ BMI < 30 and not having MetS, while MHNW was defined as BMI < 25 and not having MetS. Participants with BMI ≥30 and MetS were classified as MUO, those with 25 ≤ BMI < 30 and MetS as MUOW, and those with BMI < 25 and MetS as MUNW.

### Data analysis

The Statistical Package for the Social Sciences (SPSS) software (version 25.0, Armonk, NY: IBM Corp., USA) was used for data analysis. Mean, standard deviation, frequency, and percentages were used to describe the variables. The crude prevalence of MUO, MUOW, MUNW, MHO, MHOW, and MHNW was calculated by dividing the number of participants with each condition by 3917 (the total number of participants included in the study). Moreover, age- and sex-standardized prevalence (ASSP) was calculated through the direct standardization method using the Stata software version 15.0. Independent t-test was used to compare quantitative variables and Chi-squared test to compare qualitative variables between healthy and unhealthy states. The binary logistic regression model was used to determine the factors associated with metabolically unhealthy states. All the potential factors with *P*-values ≤0.2 in single correlations were simultaneously included in the multivariate logistic regression model using the “enter” method. For each group, the metabolically unhealthy state was coded 1 and the healthy state was coded 0. For instance, MUO was coded 1 and MHO was coded 0. Therefore, the odds of each metabolically unhealthy state were reported compared to its metabolically healthy state. *P*-values < 0.05 were regarded as statistically significant.

## Results

Of the 3917 participants included in the current study, 1691 (43.2%) were male and 2226 (56.8%) were female. Their mean age was 48.29 ± 9.39 (35–70) years. The age- and sex-standardized prevalence (ASSP) of MHO, MUO, MHOW, MUOW, MHNW and MUNW is illustrated in Table 1. Based on the criteria proposed by Meigs et al. [23], the ASSP of MHO, MHOW, and MHNW was 10.4, 22.3, and 30.3%, respectively while the ASSP of MUO, MUOW, and MUNW was found to be 13.9, 16.8, and 6.4%, respectively. The highest prevalence of MUNW (16.9%) and MUOW (25.7%) was observed in the 65–70-year age group and MUO in the 55–64-year age group (17.2%). In addition, MHNW (34%), MHOW (27.8%), and MHO (14.5%) were all the highest in the 35–44-year age group.

**Table 1:** The prevalence of MHNW, MUNW, MHOW, MUOW, MHO, and MUO based on different criteria

Criteria	Age (years)	MHNW	MUNW	MHOW	MUOW	MHO	MUO
		% (95% CI)	% (95% CI)	% (95% CI)	% (95% CI)	% (95% CI)	% (95% CI)
Meigs et al., 2006 [23]	35–44	34.0 (32.4–35.7)	2.1 (1.6–2.6)	27.8 (26.2–29.3)	10.3 (9.2–11.3)	14.5 (13.3–15.7)	11.3 (10.2–12.4)
	45–54	28.1 (26.4–29.9)	5.6 (4.6–6.5)	22.4 (20.7–24.0)	18.7 (17.1–20.2)	10.1 (8.9–11.3)	15.1 (13.7–15.5)
	55–64	27.0 (25.0–29.1)	12.6 (11.0–14.2)	14.5 (12.8–16.1)	23.8 (21.8–25.9)	4.8 (3.8–5.8)	17.2 (15.5–19.0)
	65–70	28.3 (24.3–32.3)	16.9 (13.5–20.2)	13.1 (10.1–16.1)	25.7 (21.8–29.7)	3.8 (2.1–5.5)	12.2 (9.3–15.1)
ASSP		30.3 (28.9–31.7)	6.4 (5.6–7.1)	22.3 (20.9–23.6)	16.8 (15.6–17.9)	10.4 (9.4–11.3)	13.9 (12.8–14.9)
Aguilar et al., 2008 [24]	35–44	26.4 (24.8–27.9)	9.8 (8.7–10.8)	22.4 (20.9–23.8)	15.7 (14.4–16.9)	12.7 (11.5–13.8)	13.2 (12.0–14.3)
	45–54	17.8 (16.3–19.3)	15.9 (14.4–17.4)	17.5 (16.0–19.0)	23.5 (21.8–25.2)	9.2 (8.0–10.3)	16.1 (14.6–17.5)
	55–64	15.1 (13.4–16.7)	24.6 (22.5–26.6)	9.9 (8.5–11.3)	28.4 (26.3–30.5)	4.1 (3.2–5.1)	17.9 (16.1–19.7)
	65–70	13.5 (10.4–16.6)	31.7 (27.5–35.8)	3.8 (2.1–5.5)	35.0 (30.7–39.3)	1.7 (0.5–2.8)	14.3 (11.2–17.5)
ASSP		20.4 (19.2–21.7)	16.2 (15.1–17.3)	17.0 (15.9–18.2)	22.1 (20.8–23.3)	9.1 (8.2–9.9)	15.2 (14.1–16.3)
Iranian [27]	35–44	34.2 (32.6–35.8)	1.9 (1.4–2.4)	28.3 (26.7–29.8)	9.8 (8.7–10.8)	13.4 (12.2–14.6)	12.4 (11.3–13.6)
	45–54	29.4 (27.6–31.2)	4.3 (3.5–5.1)	23.5 (21.8–25.2)	17.5 (16.0–19.0)	9.5 (8.4–10.7)	15.7 (14.3–17.2)
	55–64	27.9 (25.9–30.1)	11.6 (10.1–13.1)	13.8 (12.2–15.5)	24.5 (22.5–26.5)	4.6 (3.6–5.6)	17.5 (15.7–19.3)
	65–70	30.8 (26.7–34.9)	14.3 (11.1–17.5)	10.1 (7.4–12.8)	28.7 (24.6–32.8)	3.4 (1.8–4.9)	12.7 (9.7–15.6)
ASSP		31.1 (29.7–32.6)	5.5 (4.8–6.2)	22.5 (21.2–23.8)	16.5 (15.4–17.7)	9.7 (8.8–10.6)	14.6 (13.5–15.7)

*Abbreviations*: *ASSP* Age- and sex-standardized prevalence, *CI* Confidence interval, *MHNW* Metabolically healthy normal weight, *MUNW* Metabolically unhealthy normal weight, *MHOW* Metabolically healthy overweight, *MUOW* Metabolically unhealthy overweight, *MHO* Metabolically healthy obesity, *MUO* Metabolically unhealthy obesity

Age and weight were significantly higher in the MUNW group compared to the MHNW group. This was also the case for the MUOW group compared to the MHOW group, except for weight which was significantly higher in the MHOW group. Similarly, the aforementioned variables were higher in the MUO group compared to the MHO group; however, the difference regarding weight was not statistically significant. Daily calorie intake was surprisingly higher in the metabolically healthy groups (Table 2). In addition, the metabolically unhealthy states were most commonly observed in women, urban residents, those who were married, illiterate or unemployed, participants with moderate physical activity, and those who were at the extremes regarding wealth (the poor and the rich).

**Table 2:** Characteristics of the study population compared between healthy and unhealthy states

Variables	MHNW (*n* = 1187)	MUNW (*n* = 249)	*P*-value	MHOW (*n* = 873)	MUOW (*n* = 658)	*P*-value	MHO (*n* = 407)	MUO (*n* = 543)	*P*-value
Age (years) mean ± SD	47.37 ± 9.50	55.46 ± 8.90	< 0.001	45.90 ± 8.55	51.88 ± 8.95	< 0.001	44.57 ± 7.87	49.31 ± 8.99	< 0.001
Gender N (%)									
Male	660 (55.6)	98 (39.4)	< 0.001	467 (53.5)	194 (29.5)	< 0.001	132 (32.4)	140 (25.8)	0.025
Female	527 (44.4)	151 (60.6)		406 (46.5)	464 (70.5)		275 (67.6)	403 (74.2)	
Place of residence N (%)									
Urban	998 (84.1)	193 (77.5)	0.012	766 (87.7)	548 (83.3)	0.013	368 (90.4)	464 (85.5)	0.022
Rural	189 (15.9)	56 (22.5)		107 (12.3)	110 (16.7)		39 (9.6)	79 (14.5)	
Marital status N (%)									
Single	121 (10.2)	35 (14.1)	0.075	72 (8.2)	80 (12.2)	0.011	32 (7.9)	73 (13.4)	0.007
Married	1066 (89.8)	214 (85.9)		801 (91.8)	578 (87.8)		375 (92.1)	470 (86.6)	
Education N (%)									
Illiterate	483 (40.7)	171 (68.7)	< 0.001	276 (31.6)	384 (58.4)	< 0.001	153 (37.6)	267 (49.2)	< 0.001
literate	704 (59.3)	78 (31.3)		597 (68.4)	274 (41.6)		254 (62.4)	276 (50.8)	
Occupation N (%)									
Employed	643 (54.2)	86 (34.5)	< 0.001	500 (57.3)	195 (29.6)	< 0.001	164 (40.3)	166 (30.6)	0.002
Unemployed	544 (45.8)	163 (65.5)		373 (42.7)	463 (70.4)		243 (59.7)	377 (69.4)	
Wealth N (%)									
Poor	524 (44.1)	123 (49.4)	0.103	295 (33.8)	266 (40.4)	0.003	162 (39.8)	232 (42.7)	0.594
Average	231 (19.5)	53 (21.3)		154 (17.6)	129 (19.6)		85 (20.9)	102 (18.8)	
Rich	432 (36.4)	73 (29.3)		424 (48.6)	263 (40.0)		160 (39.3)	209 (38.5)	
Physical activity N (%)									
Low	256 (21.6)	69 (27.7)	0.019	208 (23.8)	190 (28.9)	0.004	111 (27.3)	175 (32.2)	0.092
Moderate	687 (57.9)	145 (58.2)		531 (60.8)	400 (60.8)		252 (61.9)	327 (60.2)	
High	244 (20.6)	35 (14.1)		134 (15.3)	68 (10.3)		44 (10.8)	41 (7.6)	
Weight (kg) mean ± SD	59.06 ± 8.80	60.26 ± 7.79	0.031	73.72 ± 8.84	70.91 ± 8.39	< 0.001	86.71 ± 11.42	86.78 ± 13.07	0.933
WHR mean ± SD	0.89 ± 0.06	0.96 ± 0.06	< 0.001	0.93 ± 0.05	0.97 ± 0.06	< 0.001	0.95 ± 0.06	0.97 ± 0.06	< 0.001
Daily calorie intake (kcal) mean ± SD	2879.49 ± 909.46	2581.73 ± 798.55	< 0.001	3072.19 ± 964.31	2775.48 ± 887.28	< 0.001	2992.19 ± 990.85	2867.24 ± 940.00	0.048

*Abbreviations*: *N* Number, *SD* Standard deviation, *WHR* Waist-to-hip ratio, *MHNW* Metabolically healthy normal weight, *MUNW* Metabolically unhealthy normal weight, *MHOW* Metabolically healthy overweight, *MUOW* Metabolically unhealthy overweight, *MHO* Metabolically healthy obesity, *MUO* Metabolically unhealthy obesity

Binary logistic regression analysis revealed that the adjusted odds of all metabolically unhealthy states were higher in older age groups, except for MUO whose adjusted odds were lower in the 65–70 age group compared to the 55–64 age group. Illiteracy was significantly correlated with MUOW (adjusted OR [aOR]: 1.43, 95% CI 1.09–1.87, *P* = 0.010); however, it was not associated with MUO or MUNW. Higher BMI was significantly associated with MUNW but not with MUOW or MUO. Higher WC was also significantly correlated with all metabolically unhealthy states (Table 3).

**Table 3:** Binary logistic regression analysis for the association of MUNW, MUOW, and MUO with socio-demographic characteristics

Variable	MUNW (*n* = 249) Total = 1436			MUOW (*n* = 658) Total = 1531			MUO (*n* = 543) Total = 950		
	aOR	95% CI	*P*-value	aOR	95% CI	*P*-value	aOR	95% CI	*P*-value
Gender									
Male*	1.00			1.00			1.00		
Female	1.59	0.78–3.23	0.202	1.88	1.18–2.99	0.008	1.05	0.62–1.78	0.857
Age groups (years)									
35–44*	1.00			1.00			1.00		
45–54	3.31	2.06–5.32	< 0.001	1.92	1.45–2.52	< 0.001	1.74	1.26–2.41	0.001
55–64	7.02	4.26–11.57	< 0.001	3.10	2.20–4.39	< 0.001	4.28	2.76–6.62	< 0.001
65–70	7.48	3.88–14.40	< 0.001	3.74	2.18–6.43	< 0.001	3.25	1.42–7.45	0.005
Place of residence									
Urban*	1.00			1.00			1.00		
Rural	1.10	0.74–1.63	0.655	1.10	0.79–1.54	0.561	1.37	0.88–2.15	1.166
Marital status									
Single	1.01	0.61–1.67	0.963	0.79	0.54–1.17	0.242	1.42	0.88–2.28	0.151
Married*	1.00			1.00			1.00		
Education									
Illiterate	1.26	0.84–1.88	0.260	1.43	1.09–1.87	0.010	1.04	0.74–1.46	0.824
Literate*	1.00			1.00			1.00		
Occupation									
Unemployed*	1.00			1.00			1.00		
Employed	1.22	0.79–1.90	0.376	0.75	0.55–1.02	0.068	1.01	0.67–1.53	0.972
Wealth									
Poor*	1.00			1.00			1.00		
Average	1.15	0.76–1.75	0.519	1.33	0.96–1.84	0.089	0.84	0.58–1.22	0.356
Rich	0.98	0.67–1.45	0.931	0.97	0.75–1.27	0.844	1.05	0.76–1.45	0.789
WHR									
Low*	1.00			1.00			1.00		
Moderate	0.67	0.37–1.22	0.192	1.04	0.70–1.56	0.821	1.29	0.75–2.23	0.357
High	1.08	0.52–2.24	0.833	1.17	0.72–1.89	0.526	1.32	0.75–2.32	0.342
Physical Activity									
Low	1.45	0.87–2.42	0.155	1.20	0.81–1.80	0.363	1.35	0.80–2.27	0.264
Moderate	1.16	0.74–1.82	0.513	1.20	0.84–1.72	0.312	1.35	0.83–2.19	0.226
High*	1.00			1.00			1.00		
BMI	1.20	1.07–1.36	0.003	1.01	0.92–1.11	0.801	0.96	0.91–1.02	0.202
WC	1.09	1.05–1.14	< 0.001	1.06	1.03–1.09	< 0.001	1.04	1.02–1.07	0.001

*Abbreviations*: *MUO* mEtabolically unhealthy obesity, *MUOW* Metabolically unhealthy overweight, *MUNW* Metabolically unhealthy normal weight, *aOR* Adjusted odds ratio, *CI* Confidence interval

* Reference category

## Discussion

In the current study, based on the criteria by Meigs et al. [23], the ASSP of MUO, MUOW, and MUNW was found to be 13.9, 16.8, and 6.4%, respectively. The prevalence of these metabolically unhealthy states was only slightly different using the Iranian-specific criteria [27]. However, based on the criteria by Aguilar-Salinas [24], the corresponding percentages were 15.2, 22.1, and 16.2%, respectively. This can simply be justified since meeting WC cut-offs was not required in their criteria and the cut-offs for blood pressure and FPG were set higher, leading to fewer individuals identified as metabolically unhealthy. Moreover, the prevalence of MHO, MHOW, and MHNW was 10.4, 22.3, and 30.3%, respectively in our study.

Since the 1950s, when Jean Vague observed that obese individuals’ predisposition to diabetes and atherosclerosis differs and suggested that this may be due to the difference in body fat distribution, the concept of MHO was developed [28]. There have been many studies on this subject ever since regarding BMI ≥30 kg/m^2^ as obesity and considering it a prerequisite for the definition of MHO [29]. Metabolic health has been referred to as the absence of all the components of MetS by some researchers and not meeting the criteria for the diagnosis of MetS by many others [30]. In the current study, we used the criteria proposed by Meigs et al. [23] as the basis of analysis because it was more frequently used in the literature, meanwhile reporting the prevalence of different metabolically healthy and unhealthy states according to two other sets of criteria, namely the criteria proposed by Aguilar-Salinas et al. [24] and the NCEP-ATP III criteria for MetS [25], with cut-offs for WC replaced by ≥95 cm in both men and women for the Iranian population [26].

Comparing the prevalence of MHO in the current study with what has be en reported in previous studies may not be reliable due to a lack of standardized definition. Depending on the three definitions used in our study, the ASSP prevalence of MHO ranged between 9.1 and 10.4%. Its prevalence has been demonstrated to be 4.2 to 13.6% in a Chinese adult population based on different definitions [31]. Furthermore, its prevalence has been reported to be 35% in a recent meta-analysis including 12 cohorts and 7 interventions studies [32] and approximately 12% in a collaborative analysis of 10 large cohort studies [11]. Aside from the variety of definitions, regional differences seem to contribute to these variations [11, 32]. Also, the participants of the current study were aged 35–70 years, while other studies were conducted on the adult population, usually defined as individuals over 18 years of age. Another explanation for this inconsistency might be the use of insulin resistance as a part of the diagnostic criteria proposed by Karelis et al. and Wildman et al. in some studies [33, 34].

Another finding of our study was that metabolically unhealthy states including MUO, MUOW, and MUNW were most commonly observed in women, urban residents, and those who were illiterate or unemployed. On the contrary, for metabolically healthy conditions, consisting of MHO, MHOW, and MHNW, it was the other way around; they were more frequent in men, rural residents, and those who were literate or employed. Even so, both metabolically healthy and unhealthy states were more common among those who were married, did not smoke, or were at the extremes regarding wealth (the rich and the poor). Regardless of age and gender, other factors have rarely been addressed in previous studies.

In contrast to the findings of our study, MHO has been reported to be more prevalent in women rather than men [11, 31]. This is in part due to the fact that the female participants of the current study appear to have a specific lifestyle with regard to physical activity, leading to many women falling into the MUO category; approximately 90% of the women in this study had low to moderate physical activity (data not shown). Difference in gender distribution, that is the distribution of men and women in the MHO and MUO groups of these studies compared to ours, can be another explanatory reason. The overall proportion of women could have been higher in these studies.

MHO decreased with age in our study, which is in line with the findings of van Vliet-Ostaptchouk et al. [11]. Besides, we also found that the odds of all metabolically unhealthy states elevated with increasing age, except for MUO whose odds decreased by 1.03 from the 55–64-year age group to the 65–70-year age group. A considerable variation in the prevalence of obesity phenotypes with age has been reported by Liu et al. [31]. The transient nature of MHO has been suggested by some studies including a follow-up study from England reporting the transition of 44.5% of individuals with MHO into MUO within 8 years [35]. This can partly explain the increase in the odds of metabolically unhealthy conditions with advancing age in our study.

Additionally, higher WC was significantly correlated with all metabolically unhealthy states in the current study, confirming that fat distribution and abdominal obesity are more important factors when it comes to metabolic health compared to BMI [5, 36].

Also, interestingly, we observed a significant association between BMI and MUNW but not with MUOW or MUO. This means that even when the BMI is within the normal limits (< 25 kg/m2), higher BMI is associated with increased odds of being metabolically unhealthy. Since the same correlation was not observed between BMI and MUOW or MUO, it can be speculated that although there has been no measurement of body fat in the current study, higher BMI within the normal range can translate into higher body fat in this group, which is probably not the case in overweight and obesity.

Notably, the main reason for categorization of individuals into metabolically healthy and unhealthy subgroups is to assess their cardio-metabolic risk and identify those at higher risk of cardiovascular morbidity or mortality in the long run needing special attention. Longitudinal studies best serve this purpose. Hence, a major limitation of the current study was its cross-sectional design. Besides, data regarding insulin resistance, interleukin 6 (IL-6), tumor necrosis factor-α (TNF-α), and other inflammatory markers were lacking in our study. However, the participants of this cohort are being followed and by analyzing their outcomes after 3 and 5 years of follow-up, we will be able to evaluate the significance of MHO, MHOW, or MHNW in this population. Moreover, similar to previous studies we will be able to determine the transition rate from metabolically healthy to unhealthy states and its association with patients’ outcomes.

Noteworthy, to select individuals that are most representative of the whole population in the South of Iran, Bandare-Kong city had been chosen for recruitment of the PERSIAN Cohort participants. This city has the lowest immigration and emigration rate. Meanwhile, its population is quite homogeneous with regard to lifestyle and other factors. However, we cannot determine to what extent the results of the current study reflect the true burden of metabolically unhealthy states in the whole population, which can be referred to as another limitation of the current study.

## Conclusions

Among the metabolically unhealthy conditions, the highest prevalence belonged to individuals with MUOW. Higher age and WC were significantly correlated with all metabolically unhealthy states, while illiteracy and higher BMI were only associated with MUOW and MUNW, respectively. Complementary longitudinal studies reporting the outcomes and inflammatory indices in the participants of the present study are required to confirm our findings and illustrate the significance of metabolic health in this population. Many guidelines emphasize on the significance of weight loss as a major factor to control and prevent harmful complications such as cardiovascular diseases. However, our results show that metabolic health rather than weight loss alone should be the focus and objective of public prevention programs.

## Data Availability

The datasets used and/or analyzed during the current study are available from the corresponding author on reasonable request.

## Abbreviations

ASSP: Age and sex-standardized prevalence
BMI: Body mass index
BP: Blood pressure
CI: Confidence interval
DBP: Diastolic blood pressure
FPG: Fasting plasma glucose
HC: Hip circumference
HDL: High-density lipoprotein
IL: Interleukin
METs: Metabolic equivalent of tasks
MetS: Metabolic syndrome
MHNW: Metabolically healthy normal weight
MHO: Metabolically healthy obesity
MHOW: Metabolically healthy overweight
MUNW: Metabolically unhealthy normal weight
MUO: Metabolically unhealthy obesity
MUOW: Metabolically unhealthy overweight
OR: Odds ratio
PERSIAN: Prospective Epidemiological Research Studies in IrAN
SBP: Systolic blood pressure
SPSS: Statistical Package for the Social Sciences
TG: Triglyceride
TNF: Tumor necrotizing factor
WC: Waist circumference
WHR: Waist-to-hip ratio
